# Glutamine to proline conversion is associated with response to glutaminase inhibition in breast cancer

**DOI:** 10.1186/s13058-019-1141-0

**Published:** 2019-05-14

**Authors:** Maria T. Grinde, Bylgja Hilmarsdottir, Hanna Maja Tunset, Ida Marie Henriksen, Jana Kim, Mads H. Haugen, Morten Beck Rye, Gunhild M. Mælandsmo, Siver A. Moestue

**Affiliations:** 1 Department of Circulation and Medical Imaging, Norwegian University of Science and Technology (NTNU), 7489 Trondheim, Norway; 2 Department of Tumor Biology, Institute for Cancer Research, Oslo University Hospital, Oslo, Norway; 3 Faculty of Medicine, Institute of Clinical Medicine, University of Oslo, Oslo, Norway; 4 Department of Physics, NTNU, Trondheim, Norway; 5 Department of Radiology and Nuclear Medicine, St. Olavs Hospital, Trondheim University Hospital, Trondheim, Norway; 6 Department of Cancer Research and Molecular Medicine, NTNU, Trondheim, Norway; 7 Clinic of Surgery, St. Olav’s Hospital, Trondheim University Hospital, Trondheim, Norway; 8 Institute of Medical Biology, Faculty of Health Sciences, University of Tromsø – The Arctic University of Norway, Tromsø, Norway; 9 Department of Clinical and Molecular Medicine, NTNU, Trondheim, Norway; 10 Department of Pharmacy, Nord Universitet, Namsos, Norway

**Keywords:** ^13^C MRS, Aldehyde dehydrogenase 18 family member A1 (ALDH18A1), Cancer treatment, CB-839, Gene expression analysis, Glutaminase, Glutaminase inhibitor, High-resolution magic angle spinning MR spectroscopy (HR MAS MRS), Immunohistochemistry, Patient-derived xenograft (PDX)

## Abstract

**Introduction:**

Glutaminase inhibitors target cancer cells by blocking the conversion of glutamine to glutamate, thereby potentially interfering with anaplerosis and synthesis of amino acids and glutathione. The drug CB-839 has shown promising effects in preclinical experiments and is currently undergoing clinical trials in several human malignancies, including triple-negative breast cancer (TNBC). However, response to glutaminase inhibitors is variable and there is a need for identification of predictive response biomarkers. The aim of this study was to determine how glutamine is utilized in two patient-derived xenograft (PDX) models of breast cancer representing luminal-like/ER+ (MAS98.06) and basal-like/triple-negative (MAS98.12) breast cancer and to explore the metabolic effects of CB-839 treatment.

**Experimental:**

MAS98.06 and MAS98.12 PDX mice received CB-839 (200 mg/kg) or drug vehicle two times daily p.o. for up to 28 days (*n* = 5 per group), and the effect on tumor growth was evaluated. Expression of 60 genes and seven glutaminolysis key enzymes were determined using gene expression microarray analysis and immunohistochemistry (IHC), respectively, in untreated tumors. Uptake and conversion of glutamine were determined in the PDX models using HR MAS MRS after i.v. infusion of [5-^13^C] glutamine when the models had received CB-839 (200 mg/kg) or vehicle for 2 days (*n* = 5 per group).

**Results:**

Tumor growth measurements showed that CB-839 significantly inhibited tumor growth in MAS98.06 tumors, but not in MAS98.12 tumors. Gene expression and IHC analysis indicated a higher proline synthesis from glutamine in untreated MAS98.06 tumors. This was confirmed by HR MAS MRS of untreated tumors demonstrating that MAS98.06 used glutamine to produce proline, glutamate, and alanine, and MAS98.12 to produce glutamate and lactate. In both models, treatment with CB-839 resulted in accumulation of glutamine. In addition, CB-839 caused depletion of alanine, proline, and glutamate ([1-13C] glutamate) in the MAS98.06 model.

**Conclusion:**

Our findings indicate that TNBCs may not be universally sensitive to glutaminase inhibitors. The major difference in the metabolic fate of glutamine between responding MAS98.06 xenografts and non-responding MAS98.12 xenografts is the utilization of glutamine for production of proline. We therefore suggest that addiction to proline synthesis from glutamine is associated with response to CB-839 in breast cancer.

**Graphical abstract:**

The effect of glutaminase inhibition in two breast cancer patient-derived xenograft (PDX) models. ^13^C HR MAS MRS analysis of tumor tissue from CB-839-treated and untreated models receiving ^13^C-labeled glutamine ([5-^13^C] Gln) shows that the glutaminase inhibitor CB-839 is causing an accumulation of glutamine (arrow up) in two PDX models representing luminal-like breast cancer (MAS98.06) and basal-like breast cancer (MAS98.12). In MAS98.06 tumors, CB-839 is in addition causing depletion of proline ([5-^13^C] Pro), alanine ([1-^13^C] Ala), and glutamate ([1-^13^C] Glu), which could explain why CB-839 causes tumor growth inhibition in MAS98.06 tumors, but not in MAS98.12 tumors.
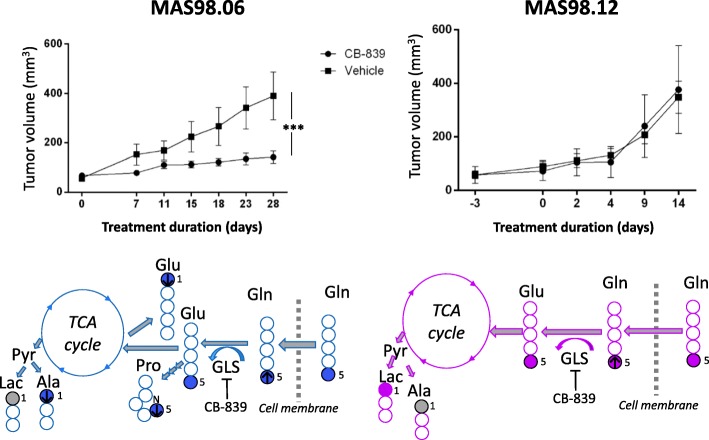

**Electronic supplementary material:**

The online version of this article (10.1186/s13058-019-1141-0) contains supplementary material, which is available to authorized users.

## Introduction

Alterations of metabolic pathways are a cancer hallmark, resulting in dependencies on specific nutrients for cell proliferation and tumor growth [1, 2]. In addition to glucose, mammalian cells use glutamine to feed the tricarboxylic acid (TCA) cycle as an alternative source of carbon, and a precursor for proteins, lipids, and nucleic acids. Glutamine is also a key precursor in the synthesis of the antioxidant glutathione, which is important in maintaining the redox balance in cells and tissues [3]. Furthermore, glutamine can be converted via glutamate to proline, which is found to play an important regulatory role in cancer [4, 5]. Recent research has identified proline as an important metabolite during adaption to hypoxia [6].

Although glutamine is a nonessential amino acid that can be synthesized from glutamate, many cancer cells depend on exogenous glutamine supply for proliferation and tumor growth [7, 8]. Upregulated glutaminolysis is observed in many aggressive forms of human cancer, including colorectal cancer [9], gliomas [10], pancreatic cancer [11], melanoma [12], and breast cancer [13, 14], which highlights the importance of this amino acid in tumor metabolism.

The rate-limiting step in glutamine metabolism is the conversion of glutamine to glutamate, which is catalyzed by the enzyme glutaminase [15]. Glutaminase exists in several tissue-specific variants, encoded by two genes in mammals, kidney-type glutaminase (*GLS1*), and liver-type glutaminase (*GLS2*). GLS1 plays a central role in tumorigenesis, whereas the role of GLS2 in cancer remains unclear. GLS1 has been found to be higher expressed in TNBC compared to other subgroups of breast cancer [14] and is essential for the survival of TNBC cells with a deregulated glutaminolysis pathway [16].

Inhibition of glutaminase has been stated as an attractive therapeutic approach in various cancers [8, 13, 14]. The glutaminase inhibitor CB-839 (Calithera) is currently being tested in clinical trials for several malignancies including breast cancer (clinicaltrials.gov, ID: NCT02071862). CB-839 is found to be specific to GLS1 and not to GLS2 [14]. Gross and colleagues found that TNBC cell lines displayed a higher sensitivity to CB-839 compared to ER+ cell lines [14]. In addition, CB-839 caused a significant antitumor activity in two selected xenograft models representing TNBC and basal-like/HER2+ breast cancer. In a clinical phase I trial, two of nine patients with TNBC treated with CB-839 experienced stabilized disease [17]. While these studies have shown promising results in the TNBC subgroup, there is a need to identify better predictive response biomarkers for optimal utilization and selection of patients that more likely will respond to CB-839.

The overall aim of this study was to identify metabolic characteristics associated with response to glutaminase inhibitors in breast cancer. First, we showed that CB-839 treatment has a differential effect on the growth of two patient-derived xenograft (PDX) models of breast cancer representing luminal-like, ER+ (MAS98.06) and basal-like, triple-negative (MAS98.12) breast cancer. Then, we investigated the potential causes of this differential response by (i) assessing the expression of selected genes and proteins directly involved in glutamine metabolism on tumor tissue from untreated mice and (ii) measuring downstream glutamine metabolites in tumor samples from CB-839-treated and untreated mice after administration of [5-^13^C] glutamine using ^13^C high-resolution magic angle spinning MR spectroscopy (HR MAS MRS).

## Materials and methods

### Animal models

The MAS98.06 and MAS98.12 patient-derived breast cancer xenograft models were established at the Institute of Cancer Research, Oslo University Hospital, as previously described [18]. The models have previously been classified as luminal B and basal-like molecular subtypes, respectively [19]. MAS98.12 has a triple-negative phenotype, whereas MAS98.06 is estrogen- and progesterone-receptor positive, and strongly dependent on estradiol supplement for tumor growth [19, 20]. Tumor tissue was bilaterally and orthotopically transplanted into 5- to 6-week-old (18–20 g) female Hsd:Athymic Nude-Foxn1^nu^ mice. The animals were kept under pathogen-free conditions at a temperature between 19 and 22 °C, humidity between 50 and 60%, 20 air changes/h, and a 12-h light/dark cycle. The animals were fed RM1 diet (Scanbur BK, Karlslunde, Denmark) and distilled tap water ad libitum. The drinking water was supplemented with 17-β-estradiol at a concentration of 4 μg/ml in order to replicate the conditions described in [18]. An overview of the experimental design is shown in Additional file 1.

All procedures and experiments involving animals were approved by the Norwegian Animal Research Authority (FOTS ID: 7713 and 9126) and carried out according to the European Convention for the Protection of Vertebrates used for Scientific Purposes.

### Tumor growth inhibition

Following bilateral transplantation, mice carrying MAS98.06 and MAS98.12 tumors (experiment 1) were kept until tumor volume reached approximately 60 mm^3^ (60.3 mm^3^ ± 32.4 mm^3^). Mice from both models were randomized to receive either CB-839 (200 mg/kg) or drug-free vehicle two times per day for up to 28 days (*n* = 5 per group). CB-839 (Calithera Biosciences, CA, USA) was dissolved in 10% cyclodextrin/saline solution and administered orally per gavage. Tumor length (l) and width (w) were measured using a digital caliper, and tumor volumes were calculated using the formula $V=\frac{1}{6}\left(\uppi \times l\times {w}^2\right)$. The mice were sacrificed at day 14 (MAS98.12) and day 28 (MAS98.06) when untreated tumors approached the upper volume limit. Untreated tumors were collected for histopathological examination and HR MAS NMR (natural abundance) analysis.

### Gene expression analysis

Gene expression analysis was performed (experiment 2) using previously generated and published data [21]. Briefly, RNA was isolated from tumor tissue from six animals from each xenograft models and hybridized to 4 × 44 k Agilent Whole Human Genome Oligo Microarrays according to the manufacturer’s protocol. The microarray data was normalized and analyzed using R(v 2.9.0) and the LIMMA Bioconductor package [22], normalized and log_2_ transformed. The microarray data is accessible through GEO Series accession number GSE37543. A total of 60 genes were selected for analysis, based on the KEGG maps central carbon metabolism in cancer (map 05230), arginine and proline biosynthesis (M00330), and glutathione biosynthesis (M00118) [23], which outline potential metabolic fates of glutamine in human cells.

Normalized and log_2_-transformed data was imported into Qlucore Omics Explorer 3.3 (Qlucore AB, Lund, Sweden) for statistical analysis. Testing for differential expression of genes between the xenograft models was performed using *t* tests with Empirical Bayesian correction of the test statistics [22]. To account for multiple testing, an adjusted *q* value of 0.05 (using Benjamini & Hochberg’s false discovery rate) was defined as the threshold for statistical significance [24].

The heatmap was generated in R (v 3.3.2) using RStudio (v 1.1.447). Hierarchical clustering was performed using the in-house made R-package Clustermap [25]. In brief, median-centered and log_2_-transformed RPPA data were clustered using Euclidean distance and complete linkage. For heatmap visualization of the data, values are normalized to the range [− 1, 1] by application of a nonlinear sigmoid transformation *f*(*x*) = tanh(*x*). This limits the visual dominance of outlier values while maintaining the order of the values, since *f* is strictly increasing.

To determine whether the metabolic characteristics of MAS98.06 and MAS98.12 xenografts are representative of the luminal B and basal-like subtypes of breast cancer, respectively, we accessed a previously published gene expression data set that in total includes 19 basal-like and 7 luminal B PDX models [19]. Gene expression of *SLC1A5*, *GLS1*, *GLUL*, and *GLUD1* was accessed and are displayed as waterfall plots in Additional file 2. The microarray data is available at the Gene Expression Omnibus (GEO) with accession number GSE44666.

### Immunohistochemistry

#### IHC staining

Seven proteins (ALDH18A1, GLS1, GLUD1, GS, Myc, PYCR1, SLC1A5) were selected for protein expression analysis based on prior knowledge on their relevance in glutaminolysis. Firstly, glutamine transporters ensure uptake of glutamine into the cells, of which neutral amino acid transporter B(0) (coded by the gene *SLC1A5* (Solute Carrier Family 1 Member 5), hereby abbreviated as SLC1A5) has received high attention since it has been shown that increased expression correlates with poor patient prognosis in many cancer types [9, 26–28]. Glutamine synthetase (GS) is the enzyme that catalyzes the conversion from glutamate to glutamine. Absence of GS is correlated with high GLS1 activity and can be associated with glutamine addiction in invasive and aggressive breast cancer phenotypes [29, 30]. Some cancer cells also rely on glutamate dehydrogenase 1 (GLUD1)-mediated Glu deamination to fuel the TCA cycle [31]. Glutamine can be converted to proline via glutamate, and the enzymes pyrroline-5-carboxylate reductase 1 (PYCR1) and aldehyde dehydrogenase 18 family member A1 (ALDH18A1) are found to be key enzymes in the conversion [6, 32, 33]. The proto-oncogene Myc is shown to be a key regulator of glutaminolysis affecting glutamine uptake, GLS1 activity, and proline metabolism [4, 26, 34–36].

Tumors from untreated mice (experiment 1) were fixed in 10% neutral-buffered formalin and embedded in paraffin. Information about primary antibodies are presented in a table in Additional file 3. GLUD1, SLC1A5, ALDH18A1, and PYCR1 staining were performed at the Cellular & Molecular Imaging Core Facility (CMIC), NTNU, Norway. GLS1, GS, and cMYC staining were performed at Covance Laboratories Inc., (Greenfield, USA). The following protocols were applied: Tumor specimens were cut into 4 μm sections which were dried, deparaffinized, and rehydrated. Heat-induced antigen retrieval was then performed for 10 (Covance, Antigen Retrieval Solution, Leica, AR9661) or 20 (CMIC, Target Retrieval Solution: Dako, low pH 6, K8005) minutes. Endogenous peroxidase activity was quenched with peroxidase block (H_2_O_2_). Sections were incubated with primary antibodies (Additional file 3) for 15 (Covance) or 40 (CMIC) mins in room temperature. Immunohistochemical reactions were visualized as specified by the vendor using either (Covance) Dako Rabbit Envision+HRP with DAB+ for use with rabbit primary antibodies (K4011, Dako) or (CMIC) Bond PDAB Reagent Kit (Leica DS9800). Sections were counterstained with hematoxylin.

#### IHC evaluation

Immunohistochemical markers were evaluated by a semiquantitative approach used to assign the histo-score (H-score) for each tumor. The H-score is given as the sum of the percentage of staining multiplied by an ordinal value corresponding to the intensity level (0 = negative, 1 = weak, 2 = moderate, 3 = strong): [1 × (% cells 1+) + 2 × (% cells 2+) + 3 × (% cells 3+)]. The final score, ranging from 0 to 300, gives more relative weight to higher intensity labeling in a given tumor sample [24, 37, 38].

An experienced pathologist from Covance Inc. scored slides labeled with antibodies against GLS1, GS, and cMYC. Two researchers (MTG and SAM) scored each slide from GLUD1, SLC1A5, ALDH18A1, and PYCR1 independently in a blinded manner. Final H-score is mean H-score ± SD from the two researchers. Some MAS98.06 tumors had central necrosis, and areas with necrosis were excluded from the analysis.

### Glutamine metabolism

Another group of mice (experiment 3) carrying MAS98.06 (*n* = 12, 30.0 ± 10.3 mm^2^) or MAS98.12 (*n* = 11, 41.4 ± 10.6 mm^2^) were randomly distributed to receive CB-839 or drug-free vehicle for 2 days as described above. Three hours after the final treatment, animals received an intravenous infusion of ^13^C-enriched glutamine (99% enrichment, Cambridge Isotope Laboratories) while under isoflurane anesthesia, as described in [39]. The mice received 1.2 mg [5-^13^C] glutamine/g body weight, dissolved in sterile PBS. A 3-min bolus of 0.3 mg/g body weight was followed by continuous infusion of 0.005 mg/g body weight/min for 180 min, in a total infusion volume of 0.8 to 1.1 ml (depending on body weight). The mice were sacrificed, and tumor tissue samples were collected, snap frozen, and stored in liquid nitrogen until NMR analysis.

### NMR experiments

#### NMR spectroscopy of tumor tissue samples

Tumor samples from both ^13^C glutamine-labeled (*n* = 5 to six per group, experiment 3) and unlabelled (N.A.: natural abundance, *n* = 3 per group, experiment 1) tumors (39.9 ± 1.1 mg) were cut to fit into a 50-μl zirconium HR MAS rotor (4-mm diameter). Lock reference containing D_2_O with formate (25 mM) was added to the rotor (~ 16 μl). The HR MAS MR spectra were recorded using a Bruker Advance DRX600 spectrometer (14.1 T) (Bruker Biospin GmbH, Germany) containing a ^1^H/^13^C MAS probe. Samples were spun at 5 kHz at magic angle, and the temperature was kept at 4 °C during the whole experiment. NMR spectra were acquired using the following NMR sequences and acquisition parameters: One-dimensional ^1^H NOESY pulse sequence with water presaturation (Bruker; noesygppr1d). Acquisition time was 2.7 s, repetition time 6.7 s, sweep width was 30 ppm, and 128 scans were acquired. The ^13^C MR spectra were acquired using a single pulse experiment, with ^1^H decoupling applied during recycle delay and acquisition (Bruker; zgpg30). The flip angle was 30°, acquisition time 0.9 s, repetition time 1.9 s, sweep width 250 ppm, and 16 k scans were obtained. Total acquisition time per sample was 9 h.

#### Analysis of NMR spectra

NMR spectra were Fourier transformed after application of line broadening (^1^H: 0.3 Hz, ^13^C: 1 Hz for tumors), and the chemical shift scale was calibrated to a reference peak ^1^H: alanine at 1.48 ppm, ^13^C: [5-^13^C] glutamine ([5-^13^C] Gln) at 180.4 ppm.

For quantification of tumor metabolites from ^1^H NMR spectra, ERETIC2 (Bruker), which is based on PULCON (PULse length-based CONcentration determination), was applied [40]. Each spectrum was scaled to sample mass, and a total of four metabolites (alanine, glutamate, glutamine, and lactate) were quantified (relative) using Chenomx software (version 8.1, Alberta, Canada). Tumor ^13^C NMR spectra were analyzed using MATLAB R2017a (The Mathworks, Inc., USA). Baseline correction was applied using an asymmetric least squares algorithm [41], and the NMR spectra were scaled to NMR sample mass. For determination of ^13^C-enriched peaks in tumors, 20 relevant peaks from the ^13^C NMR spectra were selected and integrated, and peaks that were significantly higher (Student’s *t* test, *p* < 0.05) in [5-^13^C] labeled compared to natural abundance tumors were considered ^13^C enriched. The amount of eight ^13^C-labeled metabolites in the tumors were calculated by subtracting natural abundance spectra from ^13^C-enriched spectra. The results are shown in Additional file 4 (panel c).

The different experimental groups (^13^C-enriched controls versus natural abundance controls from both models, ^13^C-enriched CB-839-treated versus ^13^C-enriched controls from both models, and ^13^C-enriched MAS98.06 controls versus ^13^C-enriched MAS98.12 controls) were compared statistically using Student’s *t* test. *P* values less than 0.05 were considered statistically significant.

## Results

### CB-839 inhibits tumor growth in the luminal-like (MAS98.06)

**Fig. 1:**
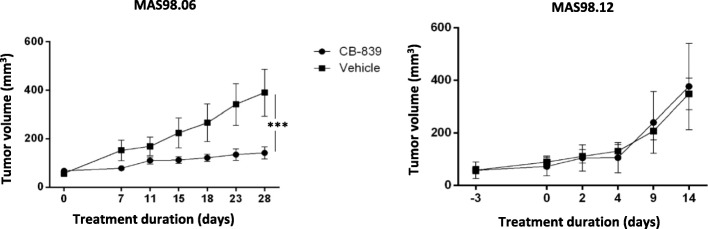
Effect of CB-839 on tumor growth in luminal-like (MAS98.06) and basal-like (MAS98.12) PDX models. CB-839 significantly (*p* < 0.001) reduces tumor growth in MAS98.06 tumors. Tumor growth of MAS98.12 breast cancer xenografts is not affected by CB-839. Mean ± SEM values are plotted. *N* = 5 for each group

Tumor growth curves from CB-839-treated and untreated MAS98.06 and MAS98.12 tumors (experiment 1) are displayed in Fig. 1 and show that CB-839 significantly inhibited the growth rate in MAS98.06 tumors. In treated mice, the tumor volume at day 28 was 28% of the volume of untreated controls (*p* = 0.0007). For the MAS98.12 model, no significant growth inhibition was observed.

### Expression of genes involved in glutaminolysis

**Fig. 2:**
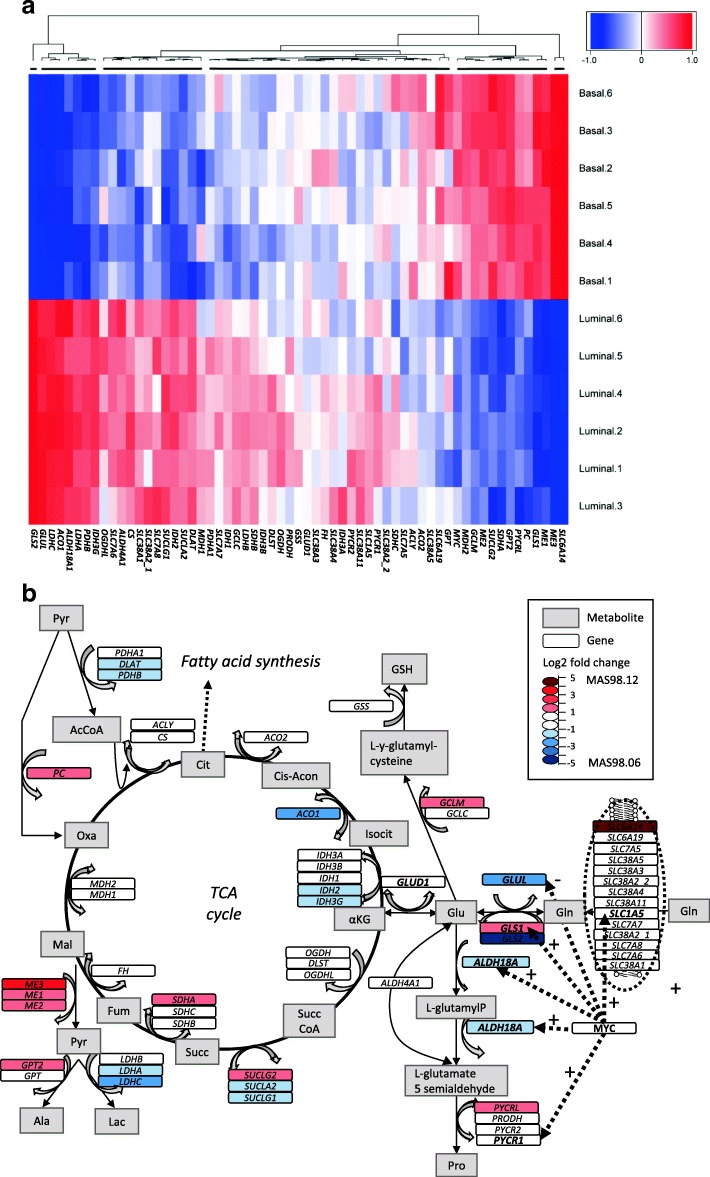
Expression of genes involved in glutaminolysis from untreated MAS98.06 and MAS98.12 tumors. **a** Heatmap of expression of the 60 selected genes involved in glutaminolysis in untreated MAS98.06 and MAS98.12 tumors. **b** Differences in gene expression between untreated MAS98.06 and MAS98.12 xenografts. Blue, the gene is significantly higher expressed with at least a two-fold higher expression in MAS98.06; red, the gene is significantly higher expressed with at least two-fold higher expression in MAS98.12; white, the gene is not significantly different expressed between the models. Log_2_-fold change (log_2_FC) between the two models is indicated by color intensity. Dotted arrows from Myc indicate some of the most important genes that are positively (+) and negatively (−) regulated by Myc, as shown by others [4, 26, 34–36]. Full gene names, enzyme commission numbers (EC numbers), gene expression levels, *q* values, log_2_ fold change, and fold changes are specified in Additional file 5

In untreated tumors (experiment 2), we observed that 31 genes were significantly higher expressed in MAS98.06 than in MAS98.12 tumors, of which 12 had at least a two-fold higher expression. For the MAS98.12 tumors, we observed that 17 genes were significantly higher expressed in this model compared to MAS98.06 tumors, of which 11 had at least a two-fold higher expression. The expression of all genes, as well as a schematic overview of genes that have at least a two-fold different expression between the models is presented in Fig. 2. A full overview of genes, expression levels and *q* values is given as Additional file 5. Among the seven key enzymes that were selected for IHC analysis, we observed that the gene expression of *GLUL* and *SLC1A5*, as well as the important genes responsible for conversion between glutamate and proline, i.e., *ALDH18A1* and *PYCR1*, were significantly more highly expressed in the responding MAS98.06 model. Contrarily, *GLS1* and *MYC* were significantly more highly expressed in MAS98.12 tumors. Of note, *SLC38A2* had two probes and the expression from both are displayed in the figure and table.

The expression of *SLC1A5*, *GLS1*, *GS*, and *GLUD1* was compared across 19 basal-like and 7 luminal B PDX models (Additional file 2). Within both basal-like and luminal B models, a considerable variability (generally five-fold to ten-fold difference) in the expression of these key genes was observed.

### Key proteins involved in glutaminolysis are differentially expressed in the two models

**Fig. 3:**
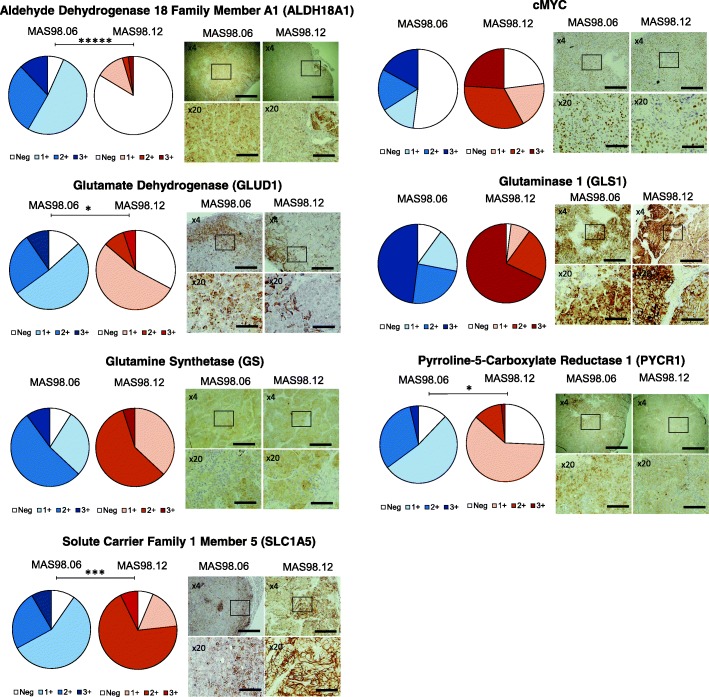
H-scores and representative images from IHC of untreated MAS98.06 and MAS98.12 tumors. The tumors were stained with antibodies against aldehyde dehydrogenase 18 family member A1 (ALDH18A1), cMYC, glutamate dehydrogenase 1 (GLUD1), glutaminase (GLS1), glutamine synthetase (GS), pyrroline-5-carboxylate reductase 1 (PYCR1), and solute carrier family 1 member 5 (SLC1A5). Left columns present the percentage areas with different intensity levels (DAB: 3′-diaminobenzidine) for MAS98.06 (blue shades), and MAS98.12 (red shades) tumors. Neg, no labeling; 1+, light label; 2+, moderate label; 3+, dark label. Right columns show representative IHC images for each antibody for both MAS98.06 tumors (left) and MAS98.12 tumors (right) in two different magnifications of × 4 and × 20. Scale bar is 200 μm for × 4 magnification and 50 μm for × 20 magnification. *p < 0.05, ***p < 0.001, *****p < 0.00001

For better understanding of the differences in glutamine utilization between the models (experiment 1), we performed immunohistochemical (IHC) analysis on selected proteins involved in glutamine metabolism in untreated tumors. Figure 3 shows percentage areas with different DAB intensity for each antibody and model in addition to representative images. Using H-score as an indicator of protein expression in the two xenograft models, MAS98.06 tumors had significantly higher expression of the two important enzymes responsible for conversion from glutamate to proline, i.e., ALDH18A1 (*p* < 1e−5), and PYCR1 (*p* < 0.05) in addition to GLUD1 (*p* < 0.05). They also had a significantly lower expression of SLC1A5 (*p* < 0.001) compared to MAS98.12 tumors. All H-scores are presented in Table 1. It was noted that SLC1A5 expression was higher in peripheral than intratumoral areas in MAS98.06 tumors, whereas MAS98.12 tumors were strongly stained throughout the tumor. A similar pattern was found for GLUD1, where MAS98.06 tumors displayed strong staining in peripheral areas and MAS98.12 tumors also displayed strong staining in intratumoral areas close to stromal cells.

**Table 1:** Histo-scores (H-score, mean ± stdev) and *p* values from selected antibodies

Protein	Histo-score		Level of significance
	MAS98.06	MAS98.12	*p* value
ALDH18A1	147 ± 25	23 ± 9	6e−06
cMYC	99 ± 52	159 ± 32	ns (0.06)
GLUD1	131 ± 35	86 ± 11	0.03
GLS1	210 ± 25	256 ± 43	ns (0.08)
GS	164 ± 25	168 ± 38	ns (0.85)
PYCR1	128 ± 24	90 ± 18	0.02
SLC1A5	132 ± 17	178 ± 6	4e−04

*Abbreviations*: *ALDH18A1* aldehyde dehydrogenase 18 family member A1, *GLUD1* glutamate dehydrogenase 1, *GLS1* glutaminase, *GS* glutamine synthetase, *ns* not significant, *PYCR1* pyrroline-5-carboxylate reductase 1, and SLC1A5 solute carrier family 1 member 5 (glutamine transporter)

### Untreated MAS98.06 and MAS98.12 tumors utilize [5-^13^C] glutamine differently

**Fig. 4:**
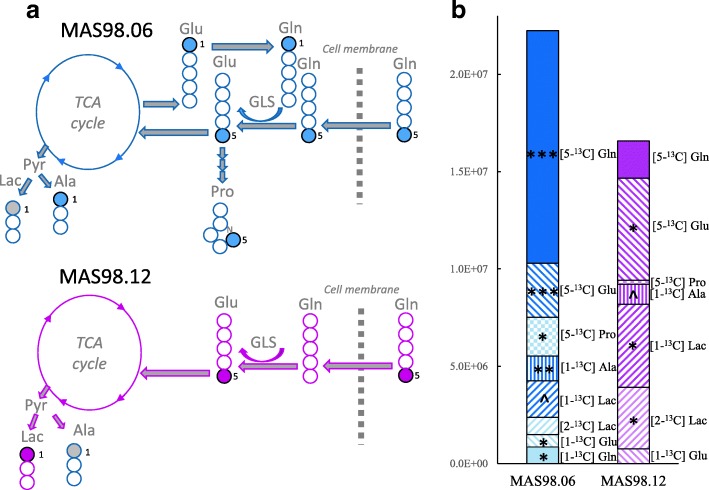
Utilization of [5-^13^C] Gln in untreated luminal-like (MAS98.06) and basal-like (MAS98.12) tumor xenografts. **a** ^13^C labeling patterns in MAS98.06 and MAS98.12 tumors after administration of [5-^13^C] Gln. MAS98.06 tumors (blue) use [5-^13^C] Gln to produce [5-^13^C] Glu, [5-^13^C] Pro, [1-^13^C] Ala, [1-^13^C] Glu, and [1-^13^C] Lac (gray, only borderline significant). They also store a significant amount of [5-^13^C] Gln in the tumors. MAS98.12 tumors (pink) take up [5-^13^C] Gln and use it for production of [5-^13^C] Glu, [1-^13^C] Lac and [5-^13^C] Ala (gray, only borderline significant). **b** Amount of ^13^C-labeled metabolites in the tumors, calculated by subtracting natural abundance spectra from ^13^C-enriched spectra. Stars (*) indicate that there is a significantly higher amount of the metabolite in ^13^C-enriched samples compared to natural abundance samples, while up arrowheads (^) indicate borderline significance. The total amount of ^13^C-labeled metabolites were not significantly different between the two models. ^*p* < 0.1, *p < 0.05, **p < 0.01, ***p < 0.001. Abbreviations: Ala, alanine; Gln, glutamine; GLS1, glutaminase; Glu, glutamate; Lac, lactate; Pro, proline; Pyr, pyruvate; TCA, tricarboxylic acid

To determine the consumption and metabolic fate of glutamine in untreated xenografts, we performed in vivo glutamine tracing experiments (experiment 3). Main findings are shown in Fig. 4. Comparing the glutamine-enriched samples with natural abundance spectra (from experiment 1), we found that the xenografts take up significant amounts of glutamine. Both models also use glutamine for anaplerotic fueling of the TCA cycle. However, while MAS98.12 tumors seem to rapidly metabolize glutamine into glutamate, lactate, and alanine, MAS98.06 tumors accumulate significant amounts of glutamine in the tumor tissue. Furthermore, MAS98.06 tumors use glutamine for production of proline, alanine, and lactate, in addition to glutamate and glutamine after one turn in the TCA cycle ([1-^13^C] Glu and [1-^13^C] Gln). A detailed presentation of ^13^C NMR spectra, in addition to ^13^C enriched and ^1^H metabolites for each group, are shown in Additional file 4. Of note, the tumor sample from one mouse with MAS98.12 xenograft had a significantly outlying ^13^C NMR spectrum, characterized with significantly higher amounts of [5-^13^C] Gln compared to the other samples in the same experimental group (Dixon’s *Q* test for outliers performed on the amount of [5-^13^C]-Glu in untreated MAS98.12 tumors showed *Q*_exp_ = 0.80 > *Q*_critCL95%_ = 0.71) [42, 43]. The samples from this mouse were removed from the results.

### CB-839 causes depletion of Pro, Ala, and Glu in the MAS98.06 model

**Fig. 5:**
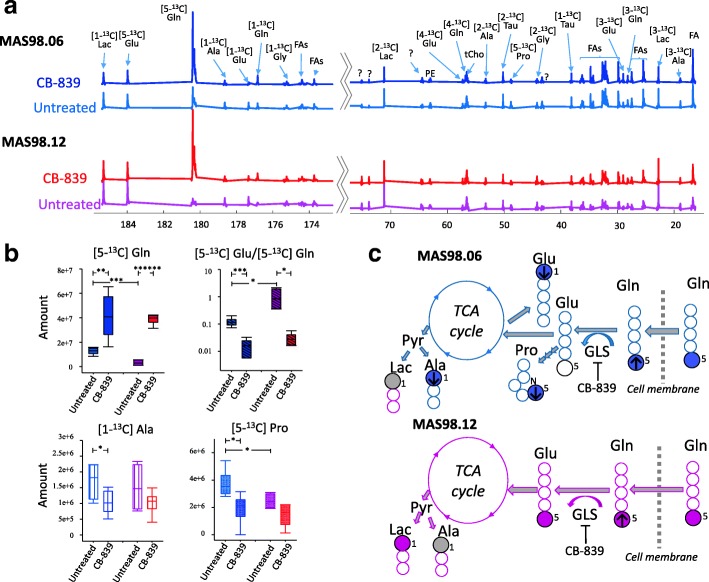
Effect of CB-839 on glutamine metabolism in MAS98.06 and MAS98.12 PDX tumors. **a** Average ^13^C NMR spectra (173.5-185.5 ppm and 75-13 ppm) for CB-839-treated and untreated MAS98.06 (blue) and MAS98.12 (red) tumors receiving ^13^C-labeled glutamine. The contribution to the average from each individual NMR spectrum is scaled to the sample mass. **b** Quantified amounts of selected ^13^C-labeled metabolites in each experimental group: ^13^C glutamine ([5-^13^C] Gln), glutamate to glutamine ratio ([5-^13^C] Glu/[5-^13^C] Gln), alanine ([1-^13^C] Ala), and proline ([5-^13^C] Pro) in the experimental groups. **c** MAS98.06 tumors take up and store glutamine (Gln) and use glutamine to produce glutamate (Glu), proline (Pro), alanine (Ala), lactate (Lac), and glutamate (Glu) through one turn in TCA cycle, as indicated by the filled blue circles (gray circle for Lac, only borderline significant). CB-839 causes an accumulation of Gln (arrow up) and reduced amounts of Pro, Al, and Glu (arrows down) in the tumors (only [1-^13^C] Glu, which is created after one turn in TCA cycle, is reduced). MAS98.12 tumors use glutamine (Gln) to produce Glu, Lac, and Ala as indicated by filled pink circles (gray circle for Ala, only borderline significant). CB-839 causes accumulation of Gln in MAS98.12 tumors, but does not significantly change the amount of any other ^13^C-enriched metabolites *p < 0.05, **p < 0.01, ***p < 0.001, ****p < 0.0001

To identify the effect of CB-839 treatment on MAS98.06 and MAS98.12 tumors, we also analyzed ^13^C NMR spectra from CB-839-treated tumors following infusion with ^13^C-labeled glutamine ([5-^13^C] Gln). Main findings from the ^13^C NMR data from treated models are shown in Fig. 5. Figure 5a shows mean ^13^C NMR spectra along with amounts of selected ^13^C-enriched metabolites (Fig. 5b) in each experimental group. A schematic overview of the most important effects of CB-839 is shown in Fig. 5c, illustrating how CB-839 caused accumulation of [5-^13^C] Gln in both tumor models, without affecting the [5-^13^C] Glu levels. In addition, it shows that CB-839 caused depletion of the downstream metabolites [5-^13^C] Pro and [1-^13^C] Ala in MAS98.06 tumors. Box plots of the amount of all ^13^C-enriched metabolites and relevant ^1^H metabolites for all experimental groups are shown in Additional file 6.

## Discussion

In this study, we have evaluated the treatment response to the glutaminase inhibitor CB-839 in two breast cancer PDX models. Previous studies suggest that basal-like and triple-negative breast cancers are more sensitive to glutaminase inhibitors than luminal-like and ER+ subgroups [14, 16]. Our findings complement this paradigm; The MAS98.06 xenografts, previously found to belong to the luminal B subtype, were sensitive to glutaminase inhibition whereas the basal-like MAS98.12 xenografts did not respond to treatment.

Previous reports have suggested that TNBC and basal-like breast cancer are particularly dependent on glutamine for proliferation and tumor growth [14, 16, 44]. Gross and colleagues showed that the TNBC subtype displayed the greatest sensitivity to CB-839 treatment in vitro and that the sensitivity was positively correlated to dependence on extracellular glutamine for growth, high baseline ratio of intracellular glutamate to glutamine (Glu:Gln), and expression of glutaminase (GLS1) [14]. A high Glu:Gln ratio has been proposed as an independent diagnostic entity. Although other studies indicate significant differences in Glu:Gln between breast cancer subgroups, a high Glu:Gln phenotype is observed across all the molecular subtypes [45]. In our experiments, we observed that the triple-negative/basal-like MAS98.12 model had significantly higher gene expression of *GLS1* and a trend of higher GLS1 protein expression, and a significantly higher Glu:Gln ratio compared to the luminal B/ER+ MAS98.06 models. Despite the accordance with previously proposed predictive biomarkers, the MAS98.12 model did not respond to CB-839 treatment, suggesting that response and resistance depend on other mechanisms regulating glutamine metabolism. Metabolic redundancy or plasticity can rescue cancer cells from glutaminase inhibition, and our understanding of metabolic characteristics within the molecular breast cancer subtypes remains limited. Several studies have demonstrated metabolic variability within subtypes and a lack of correlation between metabolic and transcriptomic traits [46, 47]. Although the triple-negative phenotype has been suggested to be particularly dependent on glutaminolysis, it has been shown that as many as 25% of these do not express GLS1 as determined by immunohistochemistry [48]. In a panel of 26 PDX models, we found more than ten-fold difference in the expression of *GLS1* within the basal-like subtype (Additional file 2). Similar variability was observed for *SLC1A5*, *GS*, and *GLUD1*. Both MAS98.06 and MAS98.12 displayed *GLS1* expression higher than the average for the cohort. No clear distinctions between basal-like and luminal B xenografts were observed for any of the genes. This indicates that although the two subtypes display metabolic differences on the population level, it must be expected that individuals within each subtype display atypical metabolic characteristics.

In order to better understand mechanisms responsible for response and resistance to glutaminase inhibitors, we studied glutamine utilization in the two xenografts by infusion of ^13^C-enriched glutamine and ex vivo ^13^C NMR tumor analysis, combined with gene- and protein expression data. From the ^13^C HR MAS MRS analysis on untreated models, we saw that the two xenograft models utilize glutamine differently. Although the total glutamine consumption was similar in the two models, the main metabolic fate of glutamine in the non-responding MAS98.12 was conversion into glutamate, lactate, and alanine. In contrast, the responding MAS98.06 tumors store significant amounts of glutamine in the tumors, but also use glutamine for synthesis of proline, alanine, and lactate. Both models use a similar fraction of the glutamine to feed the TCA cycle, as seen by the presence of [1-^13^C] glutamate (MAS98.06) and [1-^13^C] lactate (MAS98.12).

A possible scenario that may explain why some tumors are insensitive to CB-839 is that they use glycolysis instead of glutaminolysis for anaplerotic feeding of the TCA cycle. Pyruvate can enter the TCA cycle through dehydrogenation of pyruvate into acetyl-CoA (which is catalyzed by pyruvate dehydrogenase (PDH)) or through carboxylation of pyruvate into oxaloacetate (catalyzed by pyruvate carboxylase (PC)). However, we have previously found that following [1-^13^C] Glc injection, MAS98.12 tumors feed less glucose into the TCA cycle than MAS98.06 tumors, both via PDH and PC [49]. Differences in anaplerotic fueling of the TCA by glucose can therefore not explain the differences in response to CB-839 observed in this study.

It is well established that the signaling landscape plays critical roles in regulating proliferation and tumor growth [50]. The transcription factor Myc affects glutamine metabolism by enhancing glutamine uptake, glutaminase activity, and upregulation of proline metabolism [4, 26, 34–36]. Myc may trigger addiction to glutamine, which is observed both in vitro and in vivo [8, 50]. Several transporters are capable of transporting glutamine across the plasma membrane [26]. Among these, SLC1A5 has received increased attention because its expression is upregulated in many cancer types, including triple-negative breast cancer [27]. We observed that MAS98.12 xenografts display a borderline significant higher expression of MYC than MAS98.06 xenografts. This may explain the higher expression of the glutamine transporter SLC1A5 protein and *GLS1* mRNA in MAS98.12. However, expression of MYC was not associated with increased glutamine consumption or proline synthesis in our models.

In tumor tissue from the responding MAS98.06 model, we saw a depletion of proline and alanine after treatment. Alanine is produced from pyruvate, which can be synthesized using several routes, including glycolysis. Proline, on the other hand, is a conditionally essential amino acid [51]. In untreated MAS98.06 xenografts, a significant fraction of the injected [5-^13^C] glutamine was converted to [5-^13^C] proline. This is consistent with gene and protein expression data, showing that ALDH18A1 and PYCR1 were more highly expressed in MAS98.06 than MAS98.12 both on gene and protein expression level. These results indicate that MAS98.06 tumors have a higher flux from glutamate to proline compared to MAS98.12 tumors. Recent research by Tang and colleagues has identified proline as an important metabolite in cancer, linked to adaption to tumor hypoxia [6]. They showed that hypoxic microenvironments activated proline metabolism via upregulation of *ALDH18A1* in tumor samples from patients with hepatocellular carcinoma. We have previously shown that the luminal-like MAS98.06 tumors are more hypoxic than basal-like MAS98.12 tumors [20]. It could therefore be speculated that MAS98.06 tumors adapt to hypoxic microenvironments through activation of proline-mediated mechanisms. Depletion of proline following CB-839 treatment to the MAS98.06 models could leave the tumors unable to handle hypoxic stress, consequently inhibiting the growth of these tumors.

Overall, our results indicate that current biomarkers suggested for predicting response to glutaminase inhibition do not fully capture the complexity of glutamine metabolism in cancer and that the response to glutaminase inhibitors depends on the individual tumor’s ability to compensate for reduced glutamate availability. One possible explanation for the glutamine dependence in MAS98.06 tumors is that they use glutamine in proline biosynthesis, for adaption to hypoxic microenvironments.

## Conclusion

This work demonstrates that the glutaminase inhibitor CB-839, which currently is in early clinical trials for TNBC patients, significantly inhibits tumor growth in one of the breast cancer PDX models, the luminal b/ER+ MAS98.06 model. However, it had no effect on tumor growth in the other basal-like/triple-negative MAS98.12 PDX model. Gene expression analysis and IHC show that the responding model (MAS98.06) has higher gene and protein expressions of enzymes involved in the conversion of glutamine to proline. ^13^C NMR data of tissue from untreated tumors supports these findings, as the responding model has higher proline synthesis than the non-responding model. Following treatment with CB-839, ^13^C NMR demonstrated proline depletion in the responding model. One possible explanation is that the responding MAS98.06 model, which is found to be more hypoxic than non-responding MAS98.12, is dependent on proline synthesis from glutamine to adapt to the hypoxic microenvironment. Our study illustrates the shortcomings of currently proposed predictive biomarkers of response to glutaminase inhibition in breast cancer.

## Additional files

Additional file 1: Figure with an overview of the PDX models and experimental design of the study. Abbreviations: HR MAS MRS, high-resolution magic angle spinning MR spectroscopy; IHC, immunohistochemistry; N.A., natural abundance (PPTX 359 kb)

Additional file 2: Figure with gene expression of *SLC1A5*, *GLS1*, *GLUL*, and *GLUD1* in 19 basal-like and 7 luminal B PDX models. The gene expression data of *SLC1A5*, *GLS1*, *GLUL*, and *GLUD1* in 19 basal-like PDX tumors (red) and 7 basal-like PDX tumors (cyan) is presented in waterfall plots. The expressions for each genes are mean normalized. The microarray data are collected from the Gene Expression Omnibus (GEO) with accession number GSE44666 (PPTX 31009 kb)

Additional file 3: Table with overview of primary antibodies. Gives information about species reactivity, suppliers and product information, host species, and antibody dilution used during immunohistochemistry (IHC) staining (DOCX 13 kb)

Additional file 4: Figure showing HR MAS MRS data from untreated MAS98.06 and MAS98.12 tumors. a) Average ^13^C HR MAS MRS spectra calculated by subtracting natural abundance spectra from ^13^C enriched spectra. Positive signals with stars (*) indicate that there is a significantly higher amount of the metabolite in ^13^C-enriched samples compared to natural abundance samples, whereas up arrowheads (^) indicate borderline significance. b) Amount of ^13^C-labeled metabolites in the tumors, calculated by subtracting natural abundance spectra from ^13^C-enriched spectra. Stars (*) indicate that there is a significantly higher amount of the metabolite in ^13^C-enriched samples compared to natural abundance samples, and up arrowheads (^) indicate borderline significance. The total amount of ^13^C-labeled metabolites were not significantly different between the two models. c) Box plots showing the amount of the ^13^C-labeled metabolites subtracted with the amount of the metabolites from the natural abundance spectra. d) Amounts of selected metabolites from ^1^H spectra calculated from natural abundance and ^13^C-enriched samples. *p < 0.05, **p < 0.01, ***p < 0.001. Abbreviations: Ala, alanine; Gln, glutamine; GLS,: glutaminase; Glu, glutamate; Lac, lactate; Pro, proline; Pyr, pyruvate; TCA, tricarboxylic acid (PPTX 1939 kb)

Additional file 5: Table with gene expression data from the 60 selected genes. The table gives information about gene symbols, probe names, the associated EC number, *p* values and *q* values, gene expression levels for both models (log_2_ transformed), log_2_ fold change, and fold change. The table includes the same color coding system as Fig. 2 in the article. The seven selected key genes are marked in bold (DOCX 23 kb)

Additional file 6: Figure showing the effect of CB-839 in MAS98.06 and MAS98.12 tumors. a) Average ^13^C NMR spectra (173.5-185.5 ppm and 75-13 ppm) for CB-839-treated and untreated MAS98.06 and MAS98.12 models receiving ^13^C-labeled glutamine. b) Quantified amounts of ^13^C-labeled metabolites in each experimental group: ^13^C glutamine ([5-^13^C] Gln), glutamate ([5-^13^C] Glu and [1-^13^C] Glu), alanine ([1-^13^C] Ala), lactate ([1-^13^C] Lac, proline ([5-^13^C] Pro), and glutamate to glutamine ratio ([5-^13^C] Glu/[5-^13^C] Gln) in the experimental groups. c) MAS98.06 tumors take up and store glutamine (Gln) in the tumors and use glutamine to produce proline (Pro), alanine (Ala), lactate (Lac), and glutamate (Glu) through one turn in TCA cycle as indicated by filled blue circles (Lac only borderline significant, gray circle). CB-839 causes an accumulation of Gln (arrow up) and reduced amounts of Pro, Ala, and Glu (arrows down) in the tumors (only [1-^13^C] Glu, which is created after one turn in TCA cycle, is reduced). MAS98.12 tumors use glutamine (Gln) to produce Glu, Lac, and Ala as indicated by filled pink circles (Ala only borderline significant, gray circle). CB-839 causes accumulation of Gln in MAS98.12 tumors, but does not significantly change the amount of any other ^13^C-enriched metabolites. d) Quantified amount of relevant metabolites from ^1^H spectra. *p < 0.05, **p < 0.01, ***p < 0.001, ****p < 0.0001 (PPTX 339 kb)

## Abbreviations

Ala: Alanine
ALDH18A1: Aldehyde dehydrogenase 18 family member A1
DAB: 3,3′-Diaminobenzidine
ER+: Estrogen receptor positive
Gln: Glutamine
GLS1/GLS2: Glutaminase 1/glutaminase 2
Glu: Glutamate
GLUD1: Glutamate dehydrogenase 1
*GLUL*: Glutamine synthetase (gene)
GS: Glutamine synthetase (protein)
HR MAS MRS: High-resolution magic angle spinning MR spectroscopy
HRP: Horseradish peroxidase
Lac: Lactate
NMR: Nuclear magnetic resonance
PDH: Pyruvate dehydrogenase
PDX: Patient-derived xenograft
Pro: Proline
PYCR1: Pyrroline-5-carboxylate reductase 1
SLC1A5: Solute carrier family 1 member 5
*SLC38A2*: Solute carrier family 38 member 2
TNBC: Triple-negative breast cancer
