# Supplementary material for: Glutamine to proline conversion is associated with response to glutaminase inhibition in breast cancer
Source: Breast Cancer Res. 2019 May 14;21:61. doi: 10.1186/s13058-019-1141-0 (PMC6518522; doi:10.1186/s13058-019-1141-0)
Supplement: Supplementary file 1 — Figure with an overview of the PDX models and experimental design of the study. Abbreviations: HR MAS MRS, high-resolution magic angle spinning MR spectroscopy; IHC, immunohistochemistry; N.A., natural abundance (PPTX 359 kb) [file 13058_2019_1141_MOESM1_ESM.pptx]

## Slide 1
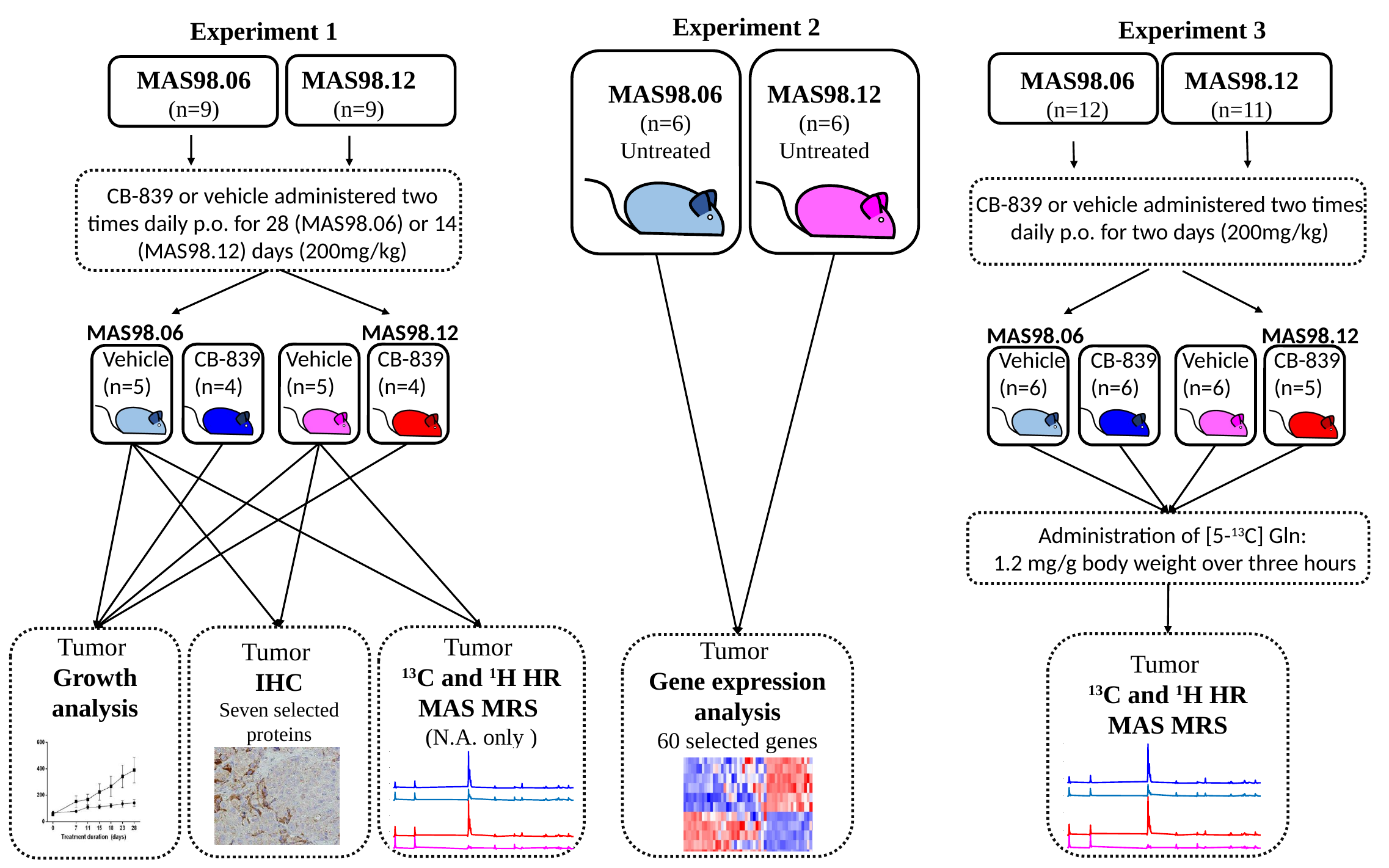

Experiment 2
MAS98.06
(n=6)
Untreated
MAS98.12
(n=6)
Untreated
Tumor
Gene expression analysis
60 selected genes
Experiment 3
MAS98.06
(n=12)
MAS98.12
(n=11)
CB-839 or vehicle administered two times daily p.o. for two days (200mg/kg)
MAS98.06		MAS98.12
Vehicle	CB-839	Vehicle	CB-839
(n=6)	(n=6)	(n=6)	(n=5)
Administration of [5-13C] Gln:
1.2 mg/g body weight over three hours
Tumor
13C and 1H HR MAS MRS
Experiment 1
MAS98.06
(n=9)
MAS98.12
(n=9)
CB-839 or vehicle administered two times daily p.o. for 28 (MAS98.06) or 14 (MAS98.12) days (200mg/kg)
MAS98.06		MAS98.12
Vehicle	CB-839	Vehicle	CB-839
(n=5)	(n=4)	(n=5)	(n=4)
Tumor
13C and 1H HR MAS MRS
(N.A. only )
Tumor
IHC
Seven selected proteins
Tumor
Growth analysis
