# Supplementary material for: Glutamine to proline conversion is associated with response to glutaminase inhibition in breast cancer
Source: Breast Cancer Res. 2019 May 14;21:61. doi: 10.1186/s13058-019-1141-0 (PMC6518522; doi:10.1186/s13058-019-1141-0)
Supplement: Supplementary file 2 — Figure with gene expression of SLC1A5, GLS1, GLUL, and GLUD1 in 19 basal-like and 7 luminal B PDX models. The gene expression data of SLC1A5, GLS1, GLUL, and GLUD1 in 19 basal-like PDX tumors (red) and 7 basal-like PDX tumors (cyan) is presented in waterfall plots. The expressions for each genes are mean normalized. The microarray data are collected from the Gene Expression Omnibus (GEO) with accession number GSE44666 (PPTX 31009 kb) [file 13058_2019_1141_MOESM2_ESM.pptx]

## Slide 1
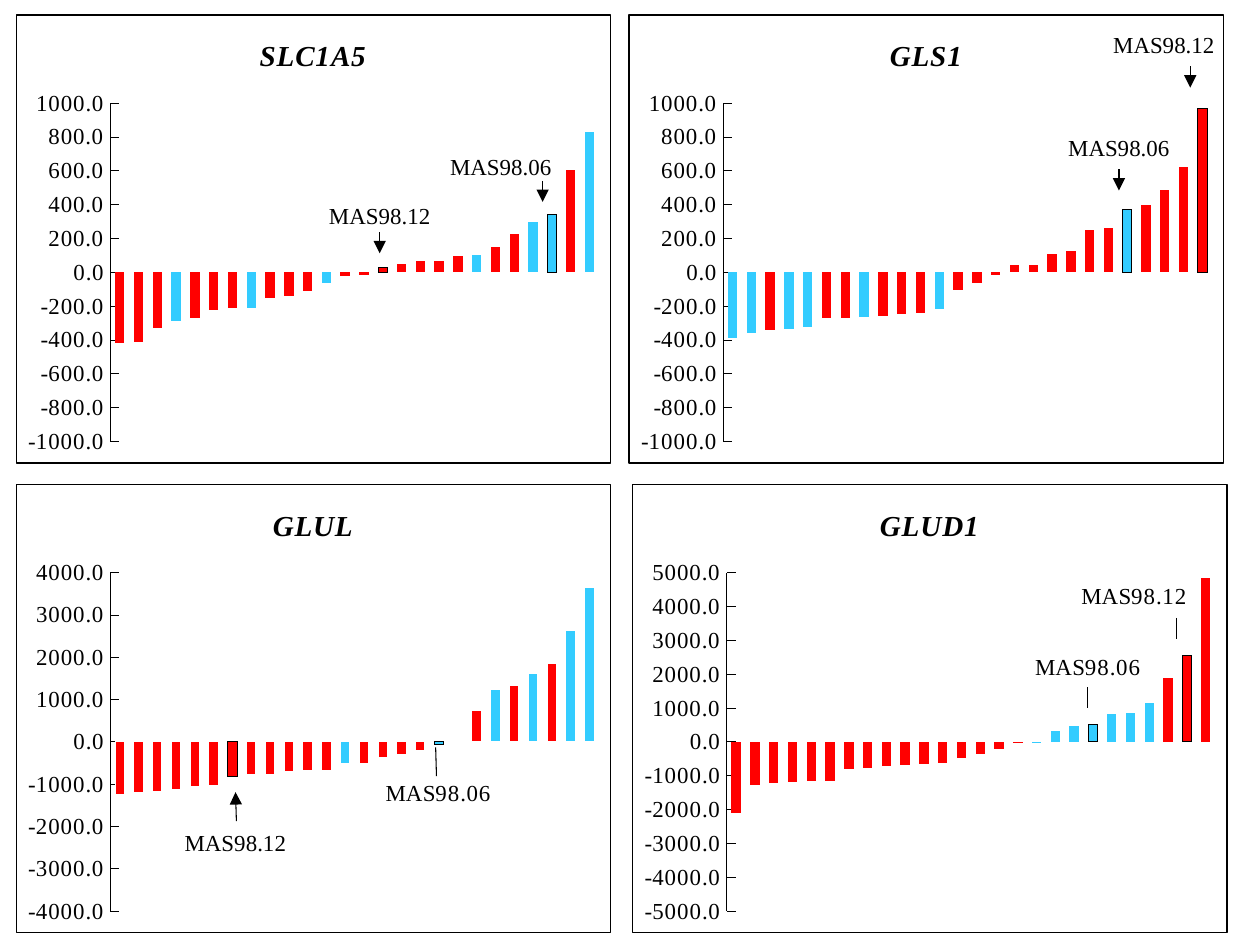

### Chart: SLC1A5
| Category | |
|---|---|MAS98.06
MAS98.12
### Chart: GLS1
| Category | |
|---|---|MAS98.12
MAS98.06
### Chart: GLUL
| Category | |
|---|---|MAS98.12
### Chart: GLUD1
| Category | |
|---|---|
