# Supplementary material for: Glutamine to proline conversion is associated with response to glutaminase inhibition in breast cancer
Source: Breast Cancer Res. 2019 May 14;21:61. doi: 10.1186/s13058-019-1141-0 (PMC6518522; doi:10.1186/s13058-019-1141-0)
Supplement: Supplementary file 3 — Table with overview of primary antibodies. Gives information about species reactivity, suppliers and product information, host species, and antibody dilution used during immunohistochemistry (IHC) staining (DOCX 13 kb) [file 13058_2019_1141_MOESM3_ESM.docx]

| **Marker** | **Antibody** | **Species reactivity** | **Supplier and catalogue no.** | **Host species** | **Antibody dilution** |
| --- | --- | --- | --- | --- | --- |
| Aldehyde dehydrogenase 18 family member A1 (ALDH18A1) | Anti-ALDH18A1 | Human, rat, mouse | Sigma Aldrich  HPA012604 | Rabbit | 1:25 |
| C-Myc | Anti-C-Myc | Mouse, rat and human | Abcam  Ab32072 | Rabbit | 1:400 |
| Glutamate dehydrogenase 1 (GLUD1) | Anti-GLUD1 | Human | Sigma Aldrich  HPA044839 | Rabbit | 1:500 |
| Glutaminase (GLS1) | Anti-GLS | Human | Abcam, ab156876 | Rabbit | 1:200 |
| Glutamine synthetase (GS) | Anti-GS | Human, mouse, rat, sheep | Millipore  MAB 302 | Mouse | 1:2000 |
| Glutamine transporter (SLC1A5) | Anti-SLC1A5 | Mouse, human, pig | Sigma Aldrich  HPA035240 | Rabbit | 1:200 |
| Pyrroline-5-carboxylate reductase 1 (PYCR1) | Anti-PYCR1 | Human | Sigma Aldrich  HPA047660 | Rabbit | 1:15 |
|  |  |  |  |  |  |
