# Supplementary material for: Glutamine to proline conversion is associated with response to glutaminase inhibition in breast cancer
Source: Breast Cancer Res. 2019 May 14;21:61. doi: 10.1186/s13058-019-1141-0 (PMC6518522; doi:10.1186/s13058-019-1141-0)
Supplement: Supplementary file 4 — Figure showing HR MAS MRS data from untreated MAS98.06 and MAS98.12 tumors. a) Average 13C HR MAS MRS spectra calculated by subtracting natural abundance spectra from 13C enriched spectra. Positive signals with stars (*) indicate that there is a significantly higher amount of the metabolite in 13C-enriched samples compared to natural abundance samples, whereas up arrowheads (^) indicate borderline significance. b) Amount of 13C-labeled metabolites in the tumors, calculated by subtracting natural abundance spectra from 13C-enriched spectra. Stars (*) indicate that there is a significantly higher amount of the metabolite in 13C-enriched samples compared to natural abundance samples, and up arrowheads (^) indicate borderline significance. The total amount of 13C-labeled metabolites were not significantly different between the two models. c) Box plots showing the amount of the 13C-labeled metabolites subtracted with the amount of the metabolites from the natural abundance spectra. d) Amounts of selected metabolites from 1H spectra calculated from natural abundance and 13C-enriched samples. *p < 0.05, **p < 0.01, ***p < 0.001. Abbreviations: Ala, alanine; Gln, glutamine; GLS,: glutaminase; Glu, glutamate; Lac, lactate; Pro, proline; Pyr, pyruvate; TCA, tricarboxylic acid (PPTX 1939 kb) [file 13058_2019_1141_MOESM4_ESM.pptx]

## Slide 1
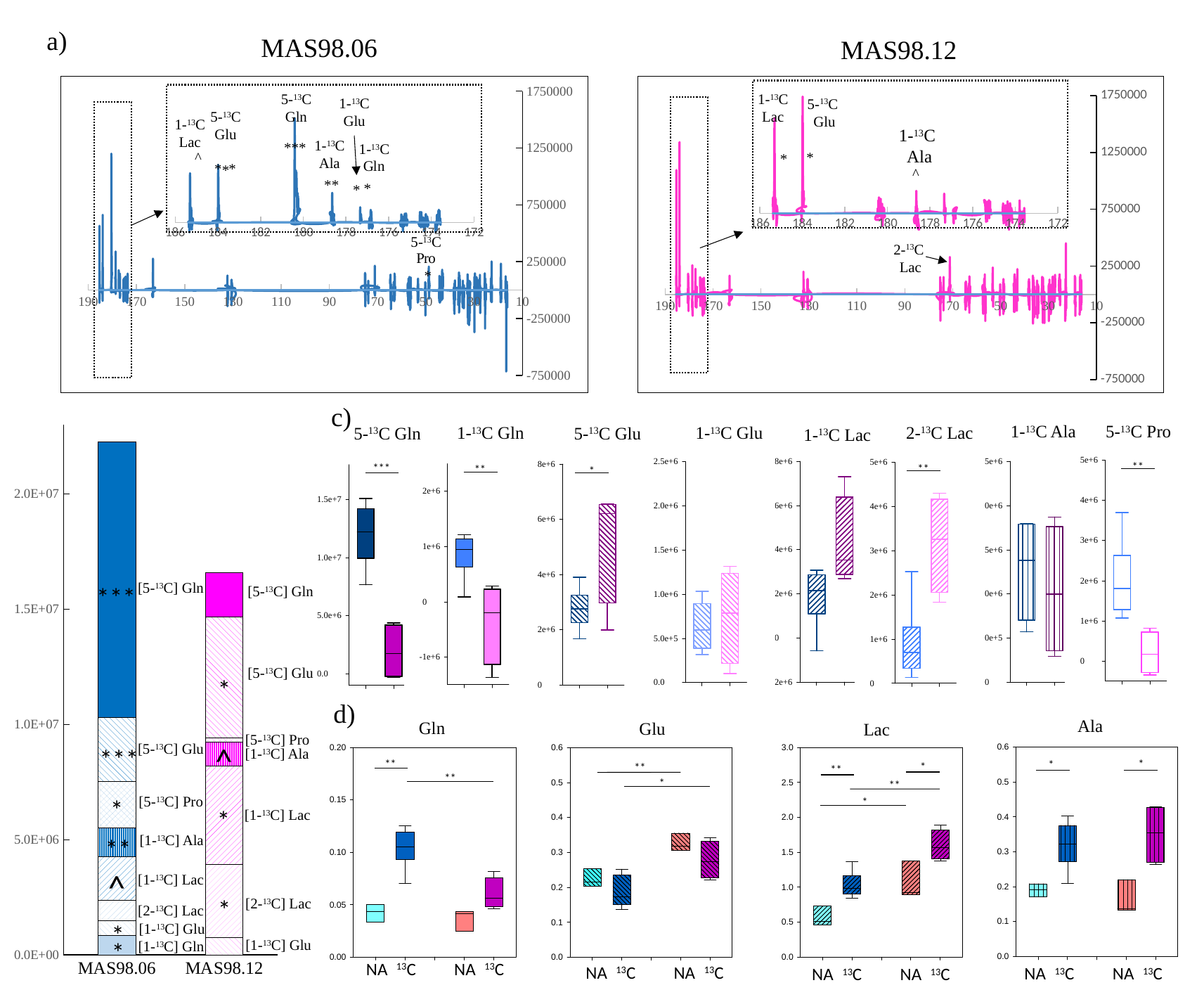

a)
MAS98.06
MAS98.12
### Chart
| Category | | MAS98.12 |
|---|---|---|
### Chart
| Category | | MAS98.12 |
|---|---|---|
1-13C
Lac
5-13C
Glu
*
*
2-13C
Lac
### Chart
| Category | | MAS98.12 |
|---|---|---|
5-13C
Gln
5-13C
Glu
1-13C
Ala
***
*
1-13C
Glu
### Chart
| Category | | MAS98.12 |
|---|---|---|*
1-13C
Lac
1-13C
Ala
^
*
### Chart
| Category | 13C-1-Gln | 13C-1-Glu | 13C-2-Lac | 13C-1-Lac | 13C-1-Ala | 13C-5-Pro | 13C-5-Glu | 13C-5-Gln |
|---|---|---|---|---|---|---|---|---|
| MAS98.06 | 852907.9277818267 | 633921.8415256784 | 889895.08576355 | 1865129.773718804 | 1276389.2096666868 | 2002049.286668998 | 2764452.007292759 | 11966258.649174333 |
| MAS98.12 | -369366.7111051079 | 748270.0974929559 | 3166838.9960323023 | 4266248.713597549 | 1040030.0964973567 | 200377.10723014147 | 5240614.694392841 | 1912485.9643994723 |[5-13C] Gln
[5-13C] Gln
[5-13C] Glu
[5-13C] Pro
[5-13C] Glu
[1-13C] Ala
[5-13C] Pro
[1-13C] Lac
[1-13C] Ala
[1-13C] Lac
[2-13C] Lac
[2-13C] Lac
[1-13C] Glu
[1-13C] Glu
[1-13C] Gln
***
*
***
*
*
**
*
*
*
c)
1-13C Ala
5-13C Pro
2-13C Lac
1-13C Glu
1-13C Gln
5-13C Glu
5-13C Gln
1-13C Lac
**
**
**
*
***
d)
Ala
Gln
Glu
Lac
^
**
*
*
*
**
**
**
*
**
*
^
NA
13C
NA
13C
NA
13C
NA
13C
NA
13C
NA
13C
NA
13C
NA
13C
