# Supplementary material for: Glutamine to proline conversion is associated with response to glutaminase inhibition in breast cancer
Source: Breast Cancer Res. 2019 May 14;21:61. doi: 10.1186/s13058-019-1141-0 (PMC6518522; doi:10.1186/s13058-019-1141-0)
Supplement: Supplementary file 5 — Table with gene expression data from the 60 selected genes. The table gives information about gene symbols, probe names, the associated EC number, p values and q values, gene expression levels for both models (log2 transformed), log2 fold change, and fold change. The table includes the same color coding system as Fig. 2 in the article. The seven selected key genes are marked in bold (DOCX 23 kb) [file 13058_2019_1141_MOESM5_ESM.docx]

| **Gene symbol** | **Probe name** | **Gene name** | **EC number** | **p-value** | **q-value** | **Average log2**  **MAS98.06** | **Average log2**  **MAS09.12** | **Log2 fold change** | **Fold change** |
| --- | --- | --- | --- | --- | --- | --- | --- | --- | --- |
| *SLC6A14* | A_23_P116614 | Solute Carrier Family 6 member 14 |  | 1.18E-09 | 1.81E-08 | 4.89 | 8.99 | 4.101 | 17.161 |
| *ME3* | A_23_P116614 | Malate dehydrogenase 3 | 1.1.1.40 | 3.69E-11 | 1.12E-09 | 5.81 | 9.64 | 3.830 | 14.221 |
| *ME1* | A_23_P422026 | Malate dehydrogenase 1 | 1.1.1.40 | 9.82E-08 | 4.51E-07 | 10.21 | 12.11 | 1.900 | 3.733 |
| ***GLS1*** | **A_23_P308800** | **Glutaminase** | **3.5.1.2** | **4.18E-09** | **4.25E-08** | **11.13** | **13.02** | **1.893** | **3.714** |
| *SDHA* | A_32_P67259 | Succinate dehydrogenase complex flavoprotein subunit A | 1.3.5.1 | 4.24E-08 | 2.35E-07 | 11.84 | 13.42 | 1.583 | 2.995 |
| *SUCLG2* | A_23_P99249 | Succinate-CoA GDP Beta subunit | 6.2.1.4/  6.2.1.5 | 1.85E-06 | 5.65E-06 | 7.87 | 9.41 | 1.544 | 2.915 |
| *GPT2* | A_24_P193648 | Alanine aminotransferase 2 | 2.6.1.2 | 1.03E-07 | 4.51E-07 | 10.06 | 11.45 | 1.395 | 2.629 |
| *PC* | A_23_P161647 | Pyruvate carboxylase | 6.4.1.1 | 5.56E-06 | 1.54E-05 | 11.13 | 12.35 | 1.220 | 2.329 |
| *ME2* | A_24_P348090 | Malate dehydrogenase 2 | 1.1.1.38 | 5.52E-07 | 1.87E-06 | 8.31 | 9.53 | 1.219 | 2.328 |
| *GCLM* | A_23_P103996 | Glutamate--cysteine ligase regulatory subunit | 6.3.2.2 | 2.56E-08 | 1.56E-07 | 8.54 | 9.71 | 1.177 | 2.261 |
| *PYCRL* | A_24_P4212 | Pyrroline-5-carboxylate reductase-Like | 1.5.1.2 | 5.84E-06 | 1.55E-05 | 12.06 | 13.20 | 1.146 | 2.213 |
| ***MYC*** | **A_23_P215956** | **MYC Proto-Oncogene** |  | **5.82E-05** | **1.18E-04** | **13.37** | **14.28** | **0.911** | **1.881** |
| *MDH2* | A_23_P31372 | Malate dehydrogenase 2 | 1.1.1.37 | 2.83E-06 | 8.21E-06 | 13.36 | 14.18 | 0.817 | 1.761 |
| *SLC6A19* | A_23_P62070 | Solute Carrier Family 6 member 19 |  | 2.03E-03 | 3.44E-03 | 7.26 | 8.07 | 0.808 | 1.751 |
| *GPT* | A_23_P146339 | Alanine aminotransferase 1 | 2.6.1.2 | 0.0374 | 0.0475 | 9.74 | 10.33 | 0.584 | 1.499 |
| *ACO2* | A_23_P103149 | Aconitase 2 | 4.2.1.3 | 1.02E-04 | 1.88E-04 | 12.76 | 13.21 | 0.454 | 1.370 |
| *ACLY* | A_23_P66787 | ATP Citrate Lyase | 2.3.3.8 | 0.0494 | 0.0614 | 10.61 | 10.85 | 0.241 | 1.182 |
| *SLC7A5* | A_24_P335620 | Solute carrier family 7 member 5 |  | 0.105 | 0.125 | 11.48 | 11.70 | 0.222 | 1.167 |
| *SLC38A5* | A_23_P84929 | Solute carrier family 38 member 5 |  | 0.0174 | 0.0241 | 5.98 | 6.18 | 0.200 | 1.148 |
| *SDHC* | A_24_P233850 | Succinate dehydrogenase complex, subunit C | 1.3.5.1 | 0.969 | 0.969 | 11.82 | 11.83 | 0.007 | 1.005 |
| *SLC38A3* | A_23_P155487 | Solute carrier family 38 member 3 |  | 0.969 | 0.969 | 5.17 | 5.17 | -0.004 | 0.997 |
| ***GLUD1*** | **A_23_P138665** | **Glutamate dehydrogenase 1** | **1.4.1.3** | **0.783** | **0.810** | **12.68** | **12.67** | **-0.018** | **0.988** |
| *SLC38A2_2* | A_32_P209173 | Solute carrier family 38 member 2 |  | 0.675 | 0.710 | 5.71 | 5.66 | -0.051 | 0.965 |
| *SLC38A4* | A_23_P25487 | Solute carrier family 38 member 4 |  | 0.660 | 0.707 | 5.02 | 4.96 | -0.054 | 0.963 |
| *GSS* | A_23_P210920 | Glutathione synthase | 6.3.2.3 | 0.465 | 0.516 | 12.14 | 12.08 | -0.061 | 0.958 |
| *IDH3A* | A_24_P927850 | Isocitrate dehydrogenase 3 alpha | 1.1.1.41 | 0.616 | 0.671 | 5.11 | 5.03 | -0.079 | 0.947 |
| *FH* | A_23_P34733 | Fumarate hydratase | 4.2.1.2 | 0.146 | 0.168 | 12.71 | 12.56 | -0.151 | 0.901 |
| *PRODH* | A_23_P68786 | Proline dehydrogenase 1 | 1.5.-.- | 0.124 | 0.146 | 5.39 | 5.22 | -0.169 | 0.890 |
| *PYCR2* | A_23_P149042 | Pyrroline-5-Carboxylate Reductase-2 | 1.5.1.2 | 0.211 | 0.239 | 10.19 | 9.99 | -0.192 | 0.875 |
| *OGDH* | A_23_P123133 | Oxoglutarate dehydrogenase like | 1.2.4.2 | 0.069 | 0.0847 | 7.99 | 7.74 | -0.246 | 0.843 |
| *SLC38A11* | A_23_P56494 | Solute carrier family 38 member 11 |  | 0.0327 | 0.0424 | 5.07 | 4.80 | -0.274 | 0.827 |
| ***PYCR1*** | **A_24_P204358** | **Pyrroline-5-Carboxylate Reductase-1** | **1.5.1.2** | **0.0207** | **0.0280** | **11.10** | **10.78** | **-0.321** | **0.800** |
| ***SLC1A5*** | **A_23_P55998** | **Solute carrier family 1 member 5** |  | **0.0169** | **0.0239** | **10.31** | **9.98** | **-0.335** | **0.793** |
| *SLC7A7* | A_23_P99642 | Solute carrier family 7 member 7 |  | 9.30E-03 | 0.0138 | 5.55 | 5.19 | -0.356 | 0.781 |
| *DLST* | A_24_P13572 | Dihydrolipoamide S-succinyltransferase | 2.3.1.61 | 0.0267 | 0.0354 | 7.46 | 7.10 | -0.357 | 0.781 |
| *IDH3B* | A_24_P936605 | Isocitrate dehydrogenase 3 beta | 1.1.1.41 | 2.70E-03 | 0.00423 | 8.18 | 7.82 | -0.359 | 0.779 |
| *PDHA1* | A_23_P251095 | Pyruvate dehydrogenase E1 alpha 1 subunit | 1.2.4.1 | 0.0118 | 0.0172 | 13.22 | 12.83 | -0.389 | 0.764 |
| *LDHB* | A_23_P53476 | Lactate dehydrogenase B | 1.1.1.27 | 9.79E-05 | 0.000188 | 16.29 | 15.84 | -0.458 | 0.728 |
| *IDH1* | A_32_P45009 | Isocitrate dehydrogenase 1 | 1.1.4.2 | 9.97E-05 | 0.000188 | 11.54 | 11.02 | -0.519 | 0.698 |
| *GCLC* | A_23_P352879 | Glutamate--cysteine ligase catalytic subunit | 6.3.2.2 | 0.000745 | 0.00134 | 8.71 | 8.17 | -0.546 | 0.685 |
| *SLC38A2_1* | A_23_P218079 | Solute carrier family 38 member 2 |  | 0.00217 | 0.00358 | 8.02 | 7.47 | -0.559 | 0.679 |
| *SDHB* | A_23_P149649 | Succinate dehydrogenase complex, subunit B | 1.3.5.1 | 2.82E-05 | 6.15E-05 | 12.37 | 11.81 | -0.559 | 0.679 |
| *MDH1* | A_23_P101950 | Malate dehydrogenase 1 | 1.1.1.37 | 0.00645 | 0.00983 | 14.21 | 13.58 | -0.634 | 0.645 |
| *SLC7A8* | A_23_P205489 | Solute carrier family 38 member 2 |  | 0.00139 | 0.00243 | 9.53 | 8.86 | -0.677 | 0.626 |
| *OGDHL* | A_23_P161297 | Oxoglutarate dehydrogenase like | 1.2.4.2 | 0.00269 | 0.00423 | 7.18 | 6.48 | -0.706 | 0.613 |
| *SLC7A6* | A_23_P88831 | Solute carrier family 7 member 6 |  | 5.80E-05 | 0.000118 | 8.62 | 7.78 | -0.839 | 0.559 |
| *CS* | A_23_P47818 | Citrate synthase | 2.3.3.1 | 1.23E-05 | 3.00E-05 | 9.13 | 8.25 | -0.879 | 0.544 |
| *SLC38A1* | A_23_P363399 | Solute carrier family 38 member 1 |  | 2.47E-05 | 5.58E-05 | 12.84 | 11.92 | -0.924 | 0.527 |
| *ALDH4A1* | A_23_P170337 | Aldehyde dehydrogenase 4 family member A1 | 1.2.1.88 | 1.40E-05 | 3.28E-05 | 11.51 | 10.52 | -0.991 | 0.503 |
| *SUCLA2* | A_23_P117157 | Succinate-CoA ADP Beta subunit | 6.2.1.4/  6.2.1.5 | 1.12E-06 | 3.59E-06 | 9.92 | 8.86 | -1.055 | 0.481 |
| *DLAT* | A_23_P203030 | Dihydrolipoamide S-acetyltransferase | 2.3.1.12 | 7.95E-06 | 2.02E-05 | 10.70 | 9.59 | -1.109 | 0.464 |
| *SUCLG1* | A_23_P79545 | Succinate-CoA GDP Alpha subunit 1 | 6.2.1.4/  6.2.1.5 | 3.25E-07 | 1.24E-06 | 11.40 | 10.14 | -1.262 | 0.417 |
| *IDH2* | A_23_P129209 | Isocitrate dehydrogenase 2 | 1.1.4.2 | 2.26E-08 | 1.53E-07 | 13.16 | 11.88 | -1.285 | 0.410 |
| *IDH3G* | A_23_P34115 | Isocitrate dehydrogenase 3 gamma | 1.1.1.41 | 5.48E-08 | 2.79E-07 | 10.63 | 9.14 | -1.486 | 0.357 |
| *LDHA* | A_32_P44568 | Lactate dehydrogenase A | 1.1.1.27 | 1.41E-07 | 5.73E-07 | 12.83 | 11.25 | -1.580 | 0.334 |
| *PDHB* | A_23_P20932 | Pyruvate dehydrogenase E1 beta subunit | 1.2.4.1 | 2.04E-08 | 1.53E-07 | 12.94 | 11.33 | -1.614 | 0.327 |
| ***ALDH18A1*** | **A_23_P1361** | **Aldehyde dehydrogenase 18 family member A1** | **2.7.2.11/ 1.2.1.41** | **3.51E-07** | **1.26E-06** | **13.25** | **11.30** | **-1.956** | **0.258** |
| *LDHC* | A_23_P53039 | Lactate dehydrogenase C | 1.1.1.27 | 2.26E-10 | 4.60E-09 | 10.40 | 7.85 | -2.546 | 0.171 |
| *ACO1* | A_23_P9416 | Aconitase 1 | 4.2.1.3 | 3.03E-09 | 3.70E-08 | 11.20 | 8.62 | -2.580 | 0.167 |
| ***GLUL*** | **A_24_P53976** | **Glutamine synthetase** | **6.3.1.2** | **1.68E-08** | **1.46E-07** | **11.62** | **8.85** | **-2.770** | **0.147** |
| *GLS2* | **A_24_P326739** | Glutaminase 2 | 3.5.1.2 | 2.91E-13 | 1.77E-11 | 10.51 | 5.56 | -4.956 | 0.032 |
