# Supplementary material for: Glutamine to proline conversion is associated with response to glutaminase inhibition in breast cancer
Source: Breast Cancer Res. 2019 May 14;21:61. doi: 10.1186/s13058-019-1141-0 (PMC6518522; doi:10.1186/s13058-019-1141-0)
Supplement: Supplementary file 6 — Figure showing the effect of CB-839 in MAS98.06 and MAS98.12 tumors. a) Average 13C NMR spectra (173.5-185.5 ppm and 75-13 ppm) for CB-839-treated and untreated MAS98.06 and MAS98.12 models receiving 13C-labeled glutamine. b) Quantified amounts of 13C-labeled metabolites in each experimental group: 13C glutamine ([5-13C] Gln), glutamate ([5-13C] Glu and [1-13C] Glu), alanine ([1-13C] Ala), lactate ([1-13C] Lac, proline ([5-13C] Pro), and glutamate to glutamine ratio ([5-13C] Glu/[5-13C] Gln) in the experimental groups. c) MAS98.06 tumors take up and store glutamine (Gln) in the tumors and use glutamine to produce proline (Pro), alanine (Ala), lactate (Lac), and glutamate (Glu) through one turn in TCA cycle as indicated by filled blue circles (Lac only borderline significant, gray circle). CB-839 causes an accumulation of Gln (arrow up) and reduced amounts of Pro, Ala, and Glu (arrows down) in the tumors (only [1-13C] Glu, which is created after one turn in TCA cycle, is reduced). MAS98.12 tumors use glutamine (Gln) to produce Glu, Lac, and Ala as indicated by filled pink circles (Ala only borderline significant, gray circle). CB-839 causes accumulation of Gln in MAS98.12 tumors, but does not significantly change the amount of any other 13C-enriched metabolites. d) Quantified amount of relevant metabolites from 1H spectra. *p < 0.05, **p < 0.01, ***p < 0.001, ****p < 0.0001 (PPTX 339 kb) [file 13058_2019_1141_MOESM6_ESM.pptx]

## Slide 1
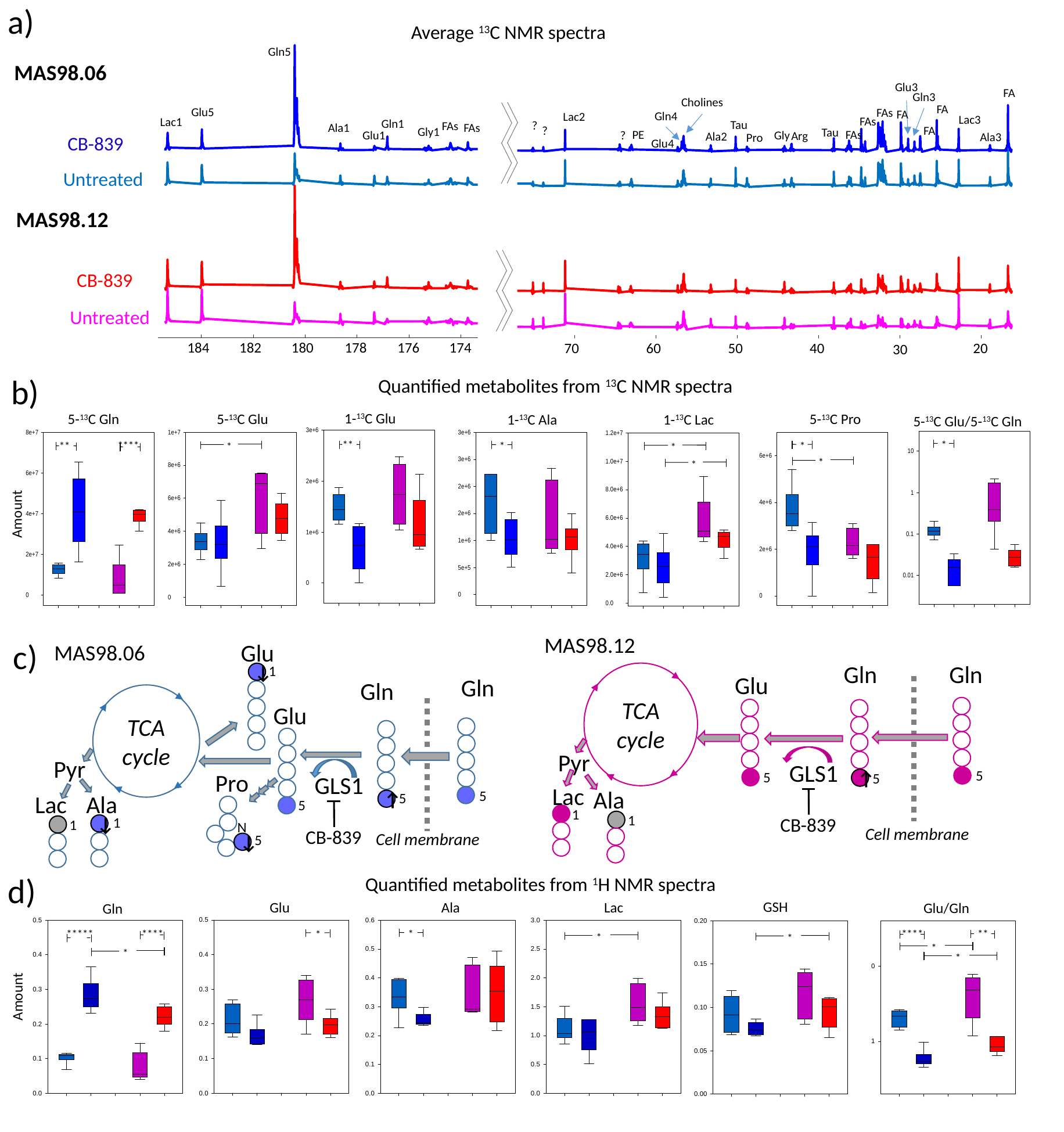

c
c
a)
Average 13C NMR spectra
Gln5
Glu5
Lac1
Gln1
FAs
Ala1
FAs
Gly1
Glu1
Glu3
FA
Gln3
Cholines
FA
FAs
FA
Gln4
Lac2
Lac3
FAs
Tau
?
FA
?
Tau
FAs
PE
?
Gly
Arg
Ala2
Ala3
Pro
Glu4
MAS98.06
CB-839
Untreated
c
MAS98.12
CB-839
Untreated
182
184
178
174
180
176
60
50
20
70
40
30
b)
Quantified metabolites from 13C NMR spectra
1-13C Glu
**
5-13C Pro
*
*
5-13C Glu
*
5-13C Gln
****
**
1-13C Lac
*
*
1-13C Ala
*
5-13C Glu/5-13C Gln
*
Amount
MAS98.12
c)
Glu
1
↓
Gln
5
Gln
5
Glu
5
Cell membrane
↑
Pyr
Pro
N
5
↓
GLS1
CB-839
Ala
↓
TCA
cycle
1
Lac
1
MAS98.06
Gln
Gln
5
TCA
cycle
Glu
5
Cell membrane
↑
Pyr
GLS1
CB-839
5
Lac
1
Ala
1
d)
Quantified metabolites from 1H NMR spectra
GSH
*
Lac
*
Ala
*
Glu
*
Glu/Gln
**
****
*
*
Gln
*****
****
*
Amount
